# *Agrobacterium* Transformation of Tea Plants (*Camellia sinensis* (L.) *KUNTZE*): A Small Experiment with Great Prospects

**DOI:** 10.3390/plants13050675

**Published:** 2024-02-28

**Authors:** Anastasia Fizikova, Elena Subcheva, Nikolay Kozlov, Varvara Tvorogova, Lidia Samarina, Ludmila Lutova, Elena Khlestkina

**Affiliations:** 1 Plant Biology and Biotechnology Department, Sirius University of Science and Technology, Olympic Avenue, 1, 354340 Sochi, Russia; subcheva.en@talantiuspeh.ru (E.S.); tvorogova.ve@talantiuspeh.ru (V.T.); subplod@mail.ru (L.L.); secretary@vir.nw.ru (E.K.); 2 Federal Research Centre the Subtropical Scientific Centre of the Russian Academy of Sciences, 2/28, Yana Fabritsiusa Street, 354002 Sochi, Russia; 3 Department of Genetics and Biotechnology, Saint Petersburg State University, Universitetskaya Emb 7/9, 199034 Saint-Petersburg, Russia; bionkbio@gmail.com; 4 N.I. Vavilov All-Russian Research Institute of Plant Genetic Resources (VIR), B. Morskaya Street, 42-44, 190000 St. Petersburg, Russia

**Keywords:** tea plant, *Agrobacterium*, transformation, transfection, protocol

## Abstract

Tea has historically been one of the most popular beverages, and it is currently an economically significant crop cultivated in over 50 countries. The Northwestern Caucasus is one of the northernmost regions for industrial tea cultivation worldwide. The domestication of the tea plant in this region took approximately 150 years, during which plantations spreading from the Ozurgeti region in northern Georgia to the southern city of Maykop in Russia. Consequently, tea plantations in the Northern Caucasus can serve as a source of unique genotypes with exceptional cold tolerance. Tea plants are known to be recalcitrant to *Agrobacterium*-mediated transfection. Research into optimal transfection and regeneration methodologies, as well as the identification of tea varieties with enhanced transformation efficiency, is an advanced strategy for improving tea plant culture. The aim of this study was to search for the optimal *Agrobacterium tumefaciens*-mediated transfection protocol for the Kolkhida tea variety. As a result of optimizing the transfection medium with potassium phosphate buffer at the stages of pre-inoculation, inoculation and co-cultivation, the restoration of normal morphology and improvement in the attachment of *Agrobacterium* cells to the surface of tea explants were observed by scanning electron microscopy. And an effective method of high-efficiency *Agrobacteria tumefaciens*-mediated transfection of the best local tea cultivar, Kolkhida, was demonstrated for the first time.

## 1. Introduction

*Camellia sinensis* is an evergreen perennial plant of the *Theaceae* family, and due to its unique metabolite composition, it has become part of the cultural heritage of many countries around the world. However, the biological characteristics of the tea tree, such as its long life cycle, extended juvenile period, and high content of secondary metabolites, place significant limitations on the improvement of cultivars through conventional breeding. The accumulation of certain metabolites, such as caffeine, tannins, and catechins—unique to tea plants—also presents challenges for plant cell cultures, particularly for the regeneration process.

Due to their prolonged life cycle, tea plants experience various stressful events, both cyclically—appearing from year to year—and associated with abrupt climate changes, which reduce the yield and quality of the harvested tea [1]. A striking example is drought, which decreases tea yield by 40% [2,3]. Other negative factors for the tea industry include high humidity, soil impoverishment with microelements, high light intensity, and cold, as well as pathogens such as *Exobasidium vexans* (“*blister blight*”) and *Helopeltis theivora* (mosquito bugs) [1,4].

Among more than 200 sequenced and released plant nuclear genomes, the tea genome is one of the most complex, primarily due to its large size, high heterozygosity, and abundance of repetitive elements [4,5]. To improve complex crops like tea, CRISPR/Cas genome editing approaches offer a faster, easier, and more precise method, with a great likelihood of preserving the valuable traits and metabolic characteristics of tea varieties [6,7]. However, *Agrobacterium*-mediated transfection is also an important tool for functional genomics in many plant crops. An important role of successful *Agrobacterium* transfection is the creation of efficient protocols for somatic embryogenesis (SE) and the regeneration of transfected explants. Tea plants are considered recalcitrant to *Agrobacterium tumefaciens*-mediated transfection (AT) [5,7,8,9]. The search for optimal transfection and regeneration methodologies, as well as the identification of tea varieties with enhanced transfection efficiency, is urgently required to allow the possibility of studying and improving tea culture [5].

Recent studies have shed light on the mechanisms of the low transfection efficiency of tea plants. It has been observed that tea leaves cause morphological abnormalities in bacterial cells and affect the attachment of the pathogen to the plant, which is a crucial step for successful *Agrobacterium* transfection. Transcriptome analyses of both the *Agrobacterium* strain GV3101 and the tea plant cultivar Bixiangzao lead to the suggestion that metabolites such as gamma-aminobutyrate and catechins—presenting in tea leaves—inhibit the attachment of *Agrobacterium*. There may also be competition for iron and potassium cations, leading to quorum defects in *Agrobacterium* cells and significantly reducing the effectiveness of the initial infection stages in tea plants [8].

In addition, the type of explant, its developmental stage, and its plant genotype, as well as the composition of the medium and the hormone combination, influence the efficiency of transfection and SE in plant species. Historically, attempts to induce tea embryogenesis began with anthers, immature cotyledon parts, immature zygotic embryos, and de-cotyledonated embryos, and later involved nodal cuttings, juvenile leaves, and petioles [1,10,11,12,13,14]. The main media used for the induction of embryogenesis were M oeS [15], WPN [16], and NN [17]. Hormone and element concentrations and incubation time varied greatly, but there was a common strategy of transitioning from media with high cytokinin content to media with low auxin content: primarily, 6-N-benzyladenine (BA) or 6-benzylaminopurine (BAP) were used in a wide range of 0 to 10 mg/L for tea; kinetin (Kn) was used at a concentration of 0.05–10 mg/L and indole-3-butyric acid (IBA) was used at 0–2 mg/L; while 1-naphthalene acetic acid (NAA), indole-3-acetic acid (IAA), and 2,4-dichlorophenoxyacetic acid (2,4-D) were used to enhance the induction of SE [9]. The use of osmoregulators—together with abscisic acid (ABA), as well as a combination of BA and gibberellic acid (GA3) and yeast extract (YE)—also contributed to increased frequency of SE [18]. Despite the presence of many tested protocols for inducing SE and transfection, the genetic differences between cultivars remain the key factor in transformation and regeneration abilities. Accordingly, the studies of tea transcriptomes and the comparison of these data with the regenerative abilities of different varieties show great importance [9].

However, several researchers have conducted genetic transformation on tea mediated by *Agrobacterium rhizogenesis* [19,20,21], as well as biolistic gun-mediated [14,22] and *Agrobacterium tumefaciens*-mediated [14,23,24,25,26] tea, using explants such as leaves [14,19,23], young shoots [21], somatic embryos [14,19,22,26], and cotyledon callus [24]. It has been shown that leaf explants are difficult to transform, as they formed stably transformed callus but failed to further differentiate [27]. Comparing all tea transformation experiments is quite difficult, as different tea varieties (cv. Kangra jat, cv. Nong Kangzao), different explants (leaves, somatic embryos, cotyledon callus, pollen callus), different *agrobacterium* strains (*A. tumefaciens* EHA105, AGL 1, *A. rhizogenes*), different transfection methods (*A. tumefaciens*/*A. rhizogenes*/Particle Gun Bombardment-mediated), and different methodologies for calculating transfection efficiency are used: some authors focus on the spots with the reporter (in most tea studies [23,24,26]), others on the number of embryos that emerge under selective conditions [28], and others calculate transfection efficiency solely based on the number of regenerated plants [14]. The latter method seems to be the most appropriate, but for woody plants that require about 8–12 months of selection and regeneration, it is time- and labor-consuming. For this reason, the formal values of transfection efficiency in tea plants vary widely, ranging from 0.5% [14] to 90% [20].

For tea plant cv. Kolkhida, micropropagation protocols were developed; however, SE and AT protocols have not been established [29,30]. The aim of this study was to search for the optimal *Agrobacterium tumefaciens*-mediated transfection protocol for the Kolkhida tea variety and semi-quantify its efficiency.

## 2. Results

**Figure 1:**
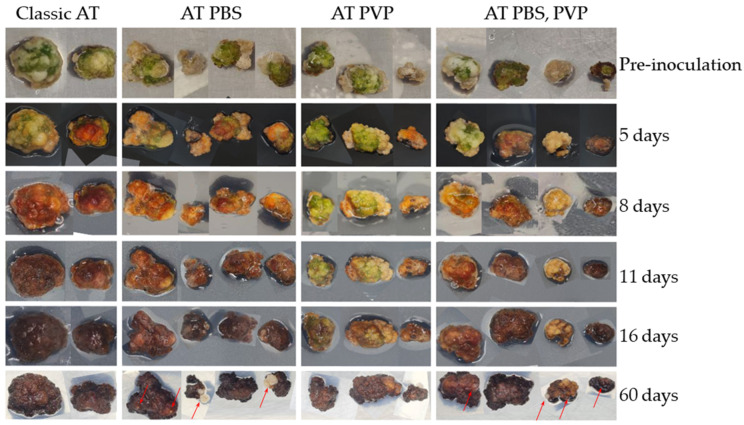
The dynamics of betalain accumulation after *AT* of cv. Kolkhida tea anther callus embryos are indicated by arrows.

To determine the efficiency of the Kolkhida tea plant variety’s AT, we conducted transfection using a vector, with the Ruby reporter and anther callus as an explant. To prepare *Agrobacterium* AGL1 cells, four different additives were applied to a co-incubation medium: (1) a control without any additives; (2) PBS buffer (pH 5.7), to a final concentration of 0.1 M; (3) PVP solution, to a final concentration of 250 mg/L; (4) a combination of both PBS and PVP in one medium; and (5) a minus control without AT (in order to evaluate the natural change in callus color due to the accumulation of phenolic metabolites). Red color indicating the accumulation of betalain became noticeable as early as the 3–5th days of observation (Figure 1).

**Figure 2:**
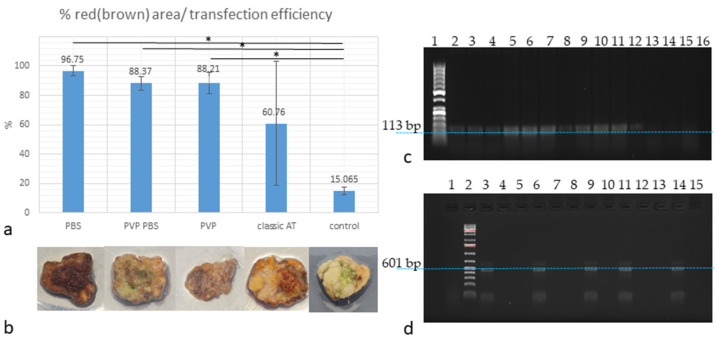
Transfection efficiency of cv. Kolkhida tea anther callus: (**a**) plot indicating percentage of transfected red/brown areas, with mean absolute percentage error, significance of proportion differences evaluated using the N1-Chi-square test: * *p* < 0.001; (**b**) Ruby-transfected calluses, from left to right: PBS, PVP PBS, PVP, classic AT, and control pieces; (**c**) electrophoregram of amplification products of Ruby transgene integrated into tea plant genome, from left to right: track 1—marker of molecular masses (1 kb Plus, Thermo Fisher Scientific Baltic, Vilnius, Lithuania); tracks 2–5—“PBS” callus; tracks 6–8—“PVP PBS” callus; tracks 9–11—“PVP”; tracks 12–15—“control” callus (1% agarose gel); (**d**) electropherogram of amplification products of the vector ori fragment, from left to right: track 1—“control” callus; track 2—marker of molecular masses (50 bp Plus, Thermo Fisher Scientific Baltic, Vilnius, Lithuania); tracks 3–6—“PBS” callus; tracks 7–9—“PVP PBS” callus; tracks 10–12—“PVP”; tracks 13–15—“control” callus (1% agarose gel). Blue-dotted lines show specific PCR product bands.

The transfection efficiency in all four experimental lines ranged from 18.6% to 100%, considering the error percentage mean. However, when transfections were conducted using the modified protocol lines, the transfection efficiency ranged from 81% to 100% (Figure 2a). Thus, the Kolkhida tea variety is transfected with high efficiency. However, when using the classical approach to prepare the agrobacterial suspension for inoculation and co-cultivation, a high variability in transfection efficiency is observed among the explants (Figure 2a).

To account for the natural accumulation of phenolic metabolites in explants and avoid confusion when interpreting the results, we performed cultivation of control callus fragments in the same media but without the addition of agrobacteria. The control calluses differed significantly in color, and there was no background accumulation of phenolic metabolites that could interfere with the result interpretation (Figure 2a,b).

After a 2-month incubation of the calluses on a regeneration medium, we extracted genomic DNA and confirmed the integration of a Ruby vector sequence into the genome using PCR (Figure 2c). Additionally, control reactions were conducted to assess the levels of agrobacteria clearance of the explants (Figure 2d). In further work, explants that did not contain any *Agrobacterium* were taken to evaluate Ruby mRNA expression level.

Remarkably, the occurrence of embryoids started on the 8th–10th days of incubation. It was observed in explants treated with *Agrobacterium* that was cultured in the presence of PBS; meanwhile, control calluses did not form embryoids even after 3 months of incubation on the regeneration medium (Figure 1). Quantification of regeneration using different AT approaches remains to be done in future experiments.

**Figure 3:**
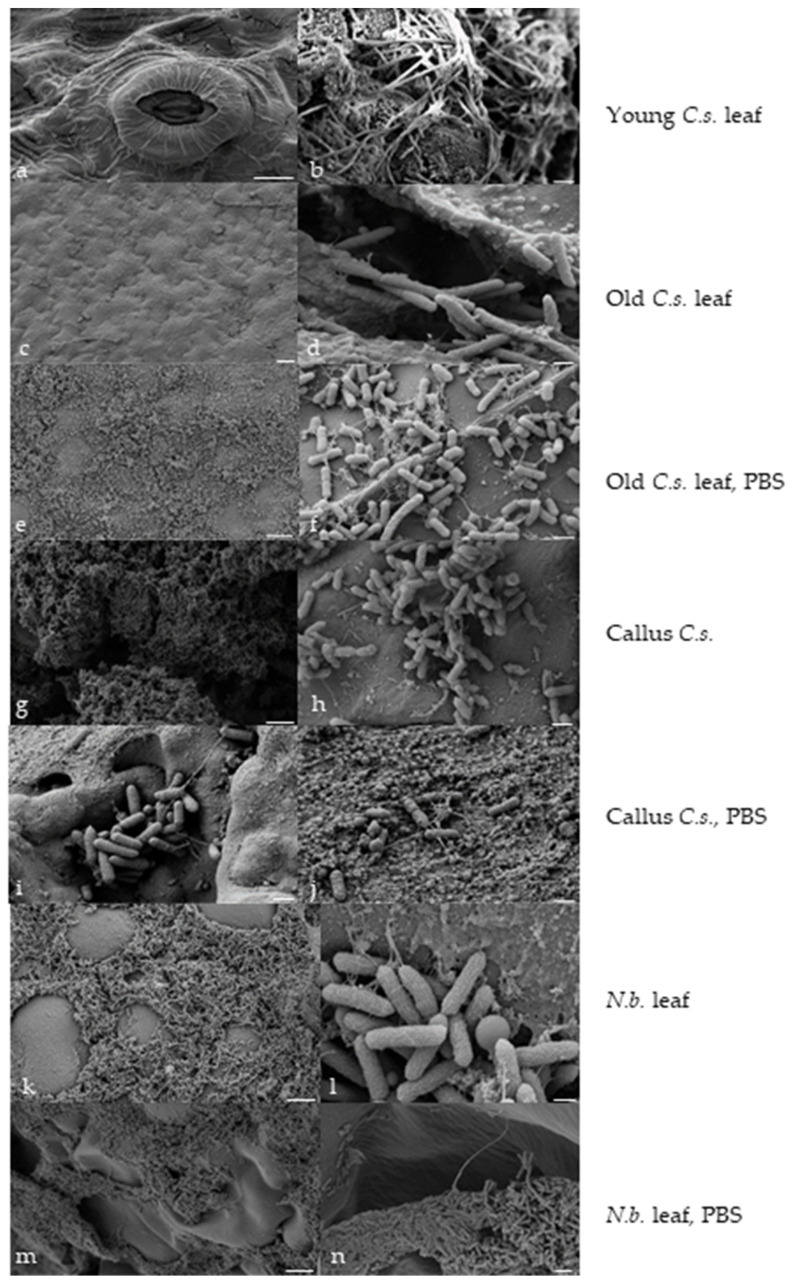
Morphology of *Agrobacterium* strain AGL1 under different inoculation conditions after transfection of tea pollen callus and leaf, SEM: (**a**) abaxial side of young leaf after AT using the standard protocol, scale bar 5 μm; (**b**) cut site of young leaf after AT using the standard protocol, scale bar 3 μm; (**c**) adaxial side of old leaf after AT using the standard protocol, scale bar 10 μm; (**d**) cut site of old leaf after AT using the standard protocol, scale bar 1 μm; (**e**) adaxial surface of old leaf after AT with inoculation using PBS, scale bar 10 μm; (**f**) surface of old leaf after AT with inoculation using PBS, scale bar 1 μm; (**g**) surface of tea callus after AT using the standard protocol, scale bar 10 μm; (**h**) surface of tea callus after AT using the standard protocol, scale bar 1 μm; (**i**) surface of tea callus after AT with inoculation using PBS, scale bar 1 μm; (**j**) surface of tea callus after AT with inoculation using PBS, scale bar 1 μm; (**k**) adaxial side of tobacco leaf after AT using the standard protocol, scale bar 10 μm; (**l**) abaxial surface of tobacco leaf after AT using the standard protocol, scale bar 500 nm; (**m**) surface of tobacco leaf after AT with inoculation using PBS, scale bar 500 nm; (**n**) surface of tobacco leaf after AT with inoculation using PBS, scale bar 2 μm.

To assess the morphology of *Agrobacterium*, their distribution, and their attachment after co-cultivation, a portion of explants was analyzed using scanning electron microscopy. As a control, tobacco (*Nicotiana benthamiana*) leaves transfected using the same *Agrobacterium* suspensions were analyzed (Figure 3). The results indicate a disruption in the attachment of *Agrobacterium* cells to the tea leaf explant: on the abaxial side of the young leaf, *Agrobacterium* cells are virtually absent (Figure 3a). Attachment of *Agrobacterium* is observed at the sites of leaf cutting; however, the cells exhibit elongated morphology, with a less dense arrangement of septa rings (Figure 3b) [31].

A disruption in the distribution and attachment of *Agrobacterium* occurred on the surfaces of the adaxial sides of the old leaves: accumulation and intertwining of *Agrobacterium* into bundles can be observed (Figure 3c,d). During *Agrobacterium* and callus inoculation with PBS, a large number of young cells connecting to each other through bridges formed a dense network that enveloped the callus and anchored *Agrobacterium* cells to the explant surface (Figure 3i,j).

The use of ordinary PBS during the pre-inoculation and inoculation stages resulted in the restoration of *Agrobacterium* cell attachment and morphology (Figure 3e,f): the cell length, formation of pilus-like structures for attachment, and plant transformation were comparable to that of the control tobacco explants (Figure 3k–n). Additionally, a large number of *Agrobacterium* cells were present in calluses, but due to the rough surface of the explant, some bacteria cells may not have been washed away by sodium chloride solution. The figures show the formation of pilus-like structures for attachment and normal cell morphology (Figure 3g,h). Moreover, a strong proliferation of *Agrobacterium* cells was observed.

**Figure 4:**
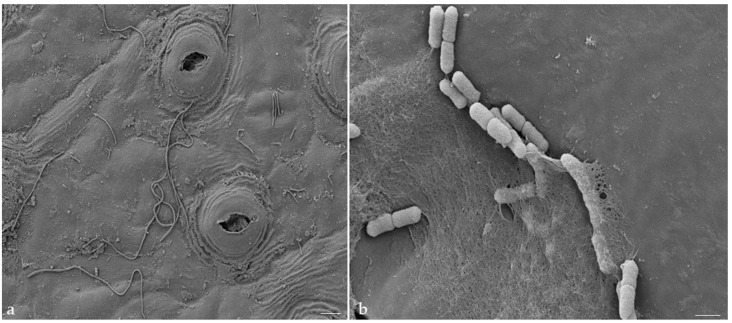
The differences in morphology of *Agrobacterium* on the abaxial and adaxial surfaces of tea leaves after inoculation with PBS, SEM: (**a**) abaxial surface of tea leaf after Agrobacterium transformation with PBS inoculation, scale bar 10 μm; (**b**) adaxial surface of tea leaf after Agrobacterium transformation with PBS inoculation, scale bar 1 μm.

The contrasting differences in *Agrobacterium* morphology on the abaxial and adaxial sides of the old tea leaves were intriguing, considering that the inoculation was performed in the same liquid medium with shaking, ensuring uniform washing of the explants with the same *Agrobacterium* cell suspension (Figure 4).

Changes in the morphology of A. tumefaciens cells and the impairment of their ability to form a bacterial film in the presence of tea metabolites, as well as a disruption of the ability of agrobacteria to attach to tea explants, may potentially explain the significant variability in the transfection efficiency of the Kolkhida tea plant using the classical AT method without the addition of PBS or PVP.

## 3. Discussion

Low AT and regeneration efficiency have significantly slowed down molecular research of tea plants. Currently, only a few successful examples of tea transformation using somatic embryos are known [26,32]. There have been no reports of successful transformation based on leaf tissue explants [23]. Although the complete genome sequence of tea plants was published six years ago [33], a deep functional analysis of tea plant genes has not been conducted due to the lack of a stable AT tea protocol. Significant efforts have been made to optimize the AT, including the use of different bacterial strains [1], various types of explants, and different co-cultivation conditions [5,25,34]. Successful transfection of leaf explants has not been achieved, nor has significant progress been made in optimizing the *Agrobacterium tumefaciens*-mediated transformation methodology as a whole.

To select optimal conditions for the transfection of plant explants, the use of the new Ruby reporter is extremely convenient. Betalain, a naturally occurring reporter, offers the distinct advantage of enabling the detection of alive transfected cells with the naked eye due to their bright red coloration [35]. Betalain is synthesized from the amino acid tyrosine through three enzymatic reactions catalyzed by L-3,4-dihydroxyphenylalanine (L-DOPA) 450 CYP76AD1 oxidase or L-DOPA-4,5-dioxygenase (DODA), forming betalamic acid, which subsequently condenses with cyclo-DOPA to form betanidin. The condensation reaction does not require enzyme participation. Following this, glucosyltransferase adds a sugar residue to betanidin, resulting in the formation of bright red betalain [36].

We have performed AT on calluses formed from different explants: leaves, anthers, axillary, and apical leaf buds. The effectiveness of the chosen regeneration strategy is yet to be assessed. The dynamics of betalain accumulation and embryo formation, which is the first stage of callus cell embryogenesis and regeneration, suggests that introducing additional sources of potassium and phosphates into the co-incubation medium promotes a faster transition of callus cells to differentiation. Calluses without *Agrobacterium*-mediated transfection were also washed with the same media and solutions as the experimental samples for control purposes. This was done because in high-metabolite plants there is a risk of not observing betalain accumulation against the background of color changes in aging tissues. However, in this case, the control samples were noticeably different and remained white–green (Figure 2b).

The *Agrobacterium* transfection of *C. sinensis* can pose certain difficulties due to several factors. Here are some of the challenges associated with this process.

1. Low transformation efficiency: The transformation efficiency, or the percentage of cells that successfully incorporate the desired gene, is relatively low. This can be attributed to various factors, such as the genotype of the tea plant, the specific tissue used for transformation, and the type of *Agrobacterium* strain employed. Low transformation efficiency can limit the number of successfully transformed plants obtained from the process [5,8,9].

2. Recalcitrant nature: *C. sinensis* is considered a recalcitrant plant species, meaning it is difficult to regenerate plants from transformed cells. The plant’s tissue culture and regeneration systems are not well-established compared to other crops, which can hinder the successful transformation and regeneration of genetically modified tea plants [5,8].

3. Tissue specificity: The type of tissue used for transformation can affect the success rate of *Agrobacterium* transformation. In *C. sinensis*, the transformation efficiency may vary depending on the tissue used, such as leaf explants or callus cultures. Selection of the optimal tissue type for transformation can be crucial, but may require extensive experiments [37,38,39,40].

4. Regeneration barriers: Regeneration of whole plants from transformed cells can be a significant challenge in *C. sinensis*. The transformed cells may not readily develop into plantlets, or may exhibit reduced growth and developmental potential. Optimizing the tissue culture and regeneration protocols specific to tea plants is essential in order to overcome these barriers [40,41,42,43,44].

During the conducted experiment, it became clear that the problem with using this transfection method for the tea plant cv Kolkhida lies not in the transfection itself, but rather in the low frequency of callus regeneration in the tea plant.

Phosphorylation plays a key role in many cellular processes, including the cell cycle; growth, apoptosis, SE, and regeneration are no exception. Thus, in *Arabidopsis*, it has previously been shown that the AtCRK5 kinase regulates the PIN2 and PIN3 proteins, influencing the levels of auxins and gibberellins through the regulation of the accumulation of polar auxin transport carriers [45]. It is known that the expression of transcription factors BBM and WUS stimulates the growth of embryogenic calluses, leading to an increase in regeneration frequency after AT in transformation-resistant monocot species [46,47,48]. Furthermore, overexpression of WUS enhances SE in dicotyledonous and gymnosperm species, such as *N. tabacum* [49], *Coffea canephora* [31], *Picea glauca* [50], and *Medicago truncatula* [28]. Also, SE receptor-like kinases (SERKs) belong to the subgroup II protein cluster of receptor-like kinases (RLK). The first SERK gene, DcSERK, was isolated from a cDNA library of carrot embryogenic cell cultures [51]. Among the various SERKs, SERK1 is highly expressed in embryogenic cultures and can be used as a reliable marker of competence for SE. During SE, SERK1 in *Arabidopsis* is widely present in all embryogenic cells and developing embryos up to the heart development stage, and its overexpression contributes to the formation of somatic embryos [51]. These effects of SERK on SE have been reported in more than one species [52] and have been associated with auxin signaling and the ability to confer pluripotency [52]. Ectopic expression of SERK1 contributes to the induction of somatic embryos, providing embryogenic competence [51], and its expression level can be used to differentiate embryogenic and non-embryogenic cells [53,54]. It can be assumed that, under conditions of competition for potassium cations during AT—as previously published [8,55]—as well as phosphates, an unfavorable situation of signal transmission inhibition may arise in the plant cell, ultimately leading to delayed embryogenesis induction.

We are not able to fully assess the regeneration frequencies now due to the specific slow dynamics of tea callus culture development, but it is already evident that when potassium phosphate buffer was added, embryos started to form on calluses as early as 8–10 days after observation began, while with the use of the standard method, nothing similar happened even after 3 months.

Similar results have been previously obtained using rice. Potassium dihydrogen phosphate was found to be essential for plant regeneration as far as no shoot regeneration occurred in the absence of an extra phosphate in the medium. The authors explained the results through the influence of potassium dihydrogen phosphate on the percent of water content in the callus [56].

The impact of potassium dihydrogen phosphate on the regenerative capacity of tea has not been previously demonstrated, which indicates the encouraging prospects of using the potassium phosphate buffer strategy in tea culture in order to enhance regenerative ability after AT.

Also, we have observed the restoration of morphology and the attachment of *Agrobacterium* cells on the surface of explants using the PBS AT protocol. The obtained results demonstrate the potential for optimizing both transfection efficiency and the frequency of SE induction and callus regeneration in tea plants through the usage of additional sources of potassium and phosphates during inoculation and co-cultivation. Previously, a decrease in the expression of the *agrobacterium* cell cycle genes *ctrA* and *divK* was shown after co-incubation with tea explants [57]. It is known that CtrA is necessary for the cell division of *A. tumefaciens* [58,59]. The activity of CtrA is controlled by an integrated pair of multicomponent phosphorelays: PleC/DivJ-DivK and CckA-ChpT-CtrA. Although some of the conservative orthologs appear to be necessary for *A. tumefaciens*. Deletions in *pleC* or *divK* have been isolated and lead to defects in cell division, decreased motility, and reduced biofilm formation. *A. tumefaciens* also has two additional sensor kinases homologous to pleC/divJ, called pdhS1 and pdhS2. Deletion of *pdhS1* creates phenocopies of *ΔpleC* and *ΔdivK* mutants. In contrast, pdhS2 may serve to regulate the switch from motility to immobility in *A. tumefaciens* [52]. It is evident that phosphorylation plays a key role in the regulation of the cell cycle and the virulence of *Agrobacterium*, which confirms the obtained results and, possibly, can explain the restoration of morphology and the attachment of *Agrobacterium* when using an additional source of phosphates and potassium in the tea AT, but this hypothesis requires further research.

The obtained results have practical significance for developing a genome editing platform for tea, in order to improve its industrial characteristics and for the functional annotation of important candidate genes.

## 4. Materials and Methods

### 4.1. Decontamination and Micropropagation of Plants

Apical and axillary buds, as well as flower buds of tea plants, were used for the initiation of in vitro culture and for callus induction. These samples were collected from the best local tea cultivar, Kolkhida, which is grown in the field collection of the Subtropical Scientific Center of the Russian Academy of Sciences.

A 10% solution of polyhexamethylene guanidine hydrochloride was used for surface decontamination of the explants for tissue culture initiation. The treatment was carried out in Falcon tubes by gently rotating at 100 rpm for 25 min, followed by washing four times with sterile distilled water and drying with sterile filter paper; then the samples were placed on the initial cultivation medium (pH 5.7) of ½ MS supplemented with 1 mg/L BAP.

The cultivation of the plants was carried out in climatic chambers at 25 °C with a light regime of 16 h of light (intensity of 70 µmol m^−2^ s^−1^) and 8 h of darkness. Callus cultures were incubated in darkness at 25 °C.

### 4.2. Callus Induction

A ½ MS medium (pH 5.7) supplemented with 5 mg/L 2,4-D was used for callus induction on leaves. The leaves were incubated in darkness and sub-cultured every 2 weeks.

A ½ MS medium (pH 5.7) supplemented with 2 mg/L 2,4-D, 1 mg/L Kn, and 1 mg/L BAP was used for callus induction on anthers [16]. The anthers were incubated in darkness and sub-cultured every two weeks. The flower buds no larger than 0.5 cm in diameter were used for anther isolation. Bright yellow anthers were isolated after sterilization and used for further experiments.

### 4.3. Vector Construction

The plasmid for Ruby overexpression was made using the GoldenGate method, with MoClo Toolkit [60] and MoClo Plant Parts Kit [61]. Ruby cds, including cds for 3 enzymes and divided by 2A peptide sequences for self-cleavage [35], was amplified from plasmid 35S:RUBY [35] and cloned into the pICH41308 vector according to the protocol [60]. Then, this cds was cloned into the pICH47751 level 1 vector [60], together with p35S double promoter from pICH51288 and t35S terminator from pICH41414 [61]. Genebank submission number: #2772079.

### 4.4. Agrobacterium Transfection of Tea Plant cv. Kolkhida

For AT, anther callus was used; 1 g of callus was weighed for each transformation. The AGL1 strain and a freeze–thaw technique transformation protocol were employed [62]. For the transfection procedure, AGL1/*Ruby* Amp+, Rf+, and PCR-confirmed *Agrobacterium* transformants were grown on YEP medium until they reached an OD 600 nm of approximately 0.4. Bacterial cells were then collected by centrifugation at 6000 rcf for 5 min and resuspended in the inoculation and co-cultivation medium (ICM). The ICM medium consisted of ½ MS (pH 5.7) with 0.44 mg/L 2,4-D, 30 g/L sucrose, 0.5 g/L glutamine, and 1 g/L casein hydrolysate, supplemented with 20 mg/L acetosyringone [63]. Four different additives were applied to the co-incubation medium: (1) a control without any additives; (2) PBS buffer (pH 5.7), to a final concentration of 0.1 M; (3) PVP solution, to a final concentration of 250 mg/L; (4) a combination of both PBS and PVP in one medium. The optical density of all four cell suspensions was adjusted to the same level of OD 0.2 after dilution. Suspensions were cultivated at room temperature (24 °C) for 1 h in order to activate Vir-gene expression by acetosyringone and to allow the agrobacteria time to adapt to the new medium. Inoculation time was 20 min. After that, the explants were rinsed with ICM and placed on semi-solid ICM medium for two days of co-cultivation in the dark at room temperature. After two days, the explants were rinsed with a liquid cocultivation medium and transferred to the ICM medium without acetosyringone, but with meropenem at a concentration of 30 mg/L to eliminate *Agrobacterium* cells. The explants were incubated at 25 °C in darkness for eight days, with sub-cultivation on fresh media and the rinsing of explants with a meropenem-containing liquid medium taking place every two days. Agrobacterial transformation was carried out in two biological and three technical replicates. The percentage of red/brown callus area (with betalain expression) was determined using ImageJ software (1.54g version) [64]. Transfection efficiency was determined as follows: the expression rate of Ruby (%) = the percentage of red or dark red area on callus surface. Regeneration efficiency was determined as the quantity of embryoids per 1 g of callus explant.

### 4.5. PCR

Ruby transgene integration was determined using transfected calluses at 60 days after inoculation. Genomic DNA was isolated using the Genomic DNA isolation kit (Norgen biotek, Thorold, ON, Canada). The primers used for PCR reaction were as follows: *prRuby-F: ATCTCCAGGCATTTCAGCCC; prRuby-R: GTGAACACCACGCCTGTAGA; prF-pUC-ORI: CTCAAGTCAGAGGTGGCGAAAC; prR-pUC-ORI: ACGTGAGTTTTCGTTCCACTGA. gDNA* was used for PCR, using the ScreenMix (Evrogen, Russia) with the Bio-Rad T100 Thermal Cycler (Biorad, Hercules, CA, USA). The PCR protocol was as follows: 95 °C for 1 min, (95 °C for 30 s, 59 °C for 20 s, 72 °C for 20 s), 30 cycles.

### 4.6. Microscopy

Small parts of leaves and calluses were washed with a 0.01 M PBS buffer (pH 5.75) and fixed with 2.5% glutaraldehyde and 4% paraformaldehyde in 0.1 M of phosphate buffer, and placed under a vacuum for 24 h. After fixation the samples were dehydrated in 30, 50, 70, 80, and 90% ethanol (for 10 min each) and two times in 95% ethanol (15 min each) at room temperature. The samples were then maintained in a mixture of 95% ethanol and isoamyl acetate (1:1) for 10 min and in pure isoamyl acetate for 15 min. We used chemical drying according to Bhattacharya et al. [65]. After the isoamyl acetate, the samples were maintained in hexamethyldisilazane for 5 min at room temperature. The samples were then dried in a desiccator for 30 min and sputter-coated with palladium/gold (20/80) (coating thickness 12 nm). The samples were viewed under a Crossbeam 550 (Carl ZEISS, QEC GmbH, München, Germany) scanning electron microscope at an accelerating voltage of 3–5 kV.

### 4.7. Statistical analysis

Statistical analyses of the data were carried out using Excel software (Microsoft^®^ Excel^®^2016(16.0.5378.1000)MSO(16.0.5366.1000)) and chi-squared tests [66,67,68].

## Data Availability

Data is contained within the article. The original contributions presented in the study are included in the article material, further inquiries can be directed to the corresponding authors.
